# Effect of exercise on pain and functional capacity in breast cancer patients

**DOI:** 10.1186/s12955-018-0882-2

**Published:** 2018-04-06

**Authors:** Andréa Dias Reis, Paula Tamara Vieira Teixeira Pereira, Renata Rodrigues Diniz, Jurema Gonçalves Lopes de Castro Filha, Alcione Miranda dos Santos, Bianca Trovello Ramallo, Florentino Assenço Alves Filho, Francisco Navarro, João Batista Santos Garcia

**Affiliations:** 1 Post-Graduate Program in Adult and Child Health, Federal University of Maranhão (Universidade Federal do Maranhão – UFMA), unit 203, street 4, n° 8, Cidade Operária, São Luis, MA Brazil; 2 Specialized Course in Sports Training (PITAGORAS), São Luís, Brazil; 3 CEUMA University, São Luís, Brazil; 4 Post-Graduate Program in Health Sciences (Federal University of Maranhão – UFMA), São Luís, Brazil; 5 Post-Graduate Program in Collective Health (Federal University of Maranhão – UFMA), São Luís, Brazil; 6 São Judas Tadeu University, São Paulo, Brazil; 7 Department of Physical Education (Federal University of Maranhão – UFMA), São Luís, Brazil

**Keywords:** Neoplasms, Exercise, Pain, Health

## Abstract

**Purpose:**

To assess the influence of combined training on pain, fatigue, maximal oxygen uptake (VO_2_ max), body mass index (BMI), flexibility, and strength in patients with breast cancer.

**Methods:**

A controlled pilot study with 28 patients undergoing chemotherapy, radiation therapy, and clinical observation in a renowned cancer treatment center; the patients were aged from 30 to 59 years old and were not engaged in physical training for three months previously. The Study Group (SG) underwent 12 weeks of training, including three 60-min sessions of aerobic exercise and resistance training, and two sessions of flexibility training per week; each flexibility exercise lasted 20 s and was performed in sets of three repetitions. The Control Group (CG) received only the standard hospital treatment. Participants were evaluated at the beginning of the study to establish a baseline and reevaluated at the end of 12 weeks.

**Results:**

Patients in the SG showed a significant decrease in total pain points (*p* = 0.0047), pain intensity (*p* = 0.0082), and the extent to which pain interfered with their daily life (*p* = 0.0047). There was an increase in maximum oxygen uptake (*p* = 0.0001), flexibility (*p* = 0.0001), and strength on both sides (right p = 0.0001 and left *p* = 0.0008). No significant differences were observed in fatigue (*p* = 0.0953) or BMI (*p* = 0.6088).

**Conclusion:**

Combined training was effective in decreasing pain and increasing VO_2_ max, flexibility and static strength in patients with breast cancer.

**Trial registration:**

NCT03061773. Registered on February 19, 2017, ‘retrospectively registered’.

## Background

Cancer accounts for more than 8 million deaths worldwide. It is estimated that the number of new cases will increase by 70% over the next two decades, making cancer one of the principal causes of morbidity and mortality. Approximately 60% of the new cases of cancer occur each year in Africa, Asia, Central and South America; these regions are also responsible for the majority of cancer deaths [37]. Estimates for 2016–2017 predict 596,000 cases of cancer in Brazil, of which 300,800 are expected to be women, including 57,960 cases of breast cancer [27].

Treatment for breast cancer is expensive: it costs more than $ 13.89 for a single patient to undergo biopsies, sectorectomy, chemotherapy (taxanes followed by anthracycline), radiation therapy, and 5 years of tamoxifen. These costs can be even higher depending on the treatment regimen, making breast cancer a public health problem [17].

The adverse side effects of cancer treatment include pain, fatigue, cachexia (wasting syndrome), diminished strength and lung capacity, and reduced range of movement, among others [7, 15, 21, 29]. Six months after diagnosis, approximately 90% of women manifested at least one adverse side effect from cancer treatment, while 60% experienced multiple late effects that influenced their treatment and quality of life, and consequently their survival rates. Furthermore, 6 years after treatment, 30% of women reported treatment-related late effects, which have implications for morbidity and mortality rates [32].

Pain is one of the most common symptoms in patients with breast cancer, with 30–60% experiencing moderate to intense pain [13]. In addition to the high prevalence of pain among these patients, approximately half of them receive inadequate treatment; this may be related to a failure in identifying the intensity of pain, or underestimation of the severity of pain.

Pain tends to decrease by physical training, which increases strength, cardiorespiratory fitness, flexibility, and quality of life, and decreases fatigue, length of hospital stay, anxiety, depression, stress, sleep disorders, nausea, and vomiting. It also contributes to better responses to treatment, body image, mood, and body mass maintenance (maintaining or increasing muscle tissue and reducing body fat) [15, 19, 23].

In a multi-centric study with 301 patients undergoing chemotherapy, it was observed that higher-intensity aerobic training was more effective at decreasing pain, compared to lower-intensity aerobic training and combined training. However, pain assessment in the previous study was performed by using a quality-of-life questionnaire rather than using a specific scale [8].

Combined training has been studied as a means for decreasing pain in patients with breast cancer. One study demonstrated that combined training, which included aerobic and resistance exercises for eight months, succeeded in decreasing pain [15]. Another study showed that while combined aquatic exercise training for 8 weeks, which included aerobic, resistance, and flexibility exercises, reduced pain, they did not improve sure muscle pain, and even increased cervical spine (neck) pain [7]. Due to the scarcity of random clinical trials evaluating patients’ pain, a physical training protocol that outlines the ideal duration, intensity, and combination of different exercises in a single session has yet to be established for the treatment of pain in patients with breast cancer [3, 6].

The present study aims to evaluate the influence of a 12-week course of combined training containing aerobic, resistance, and flexibility exercises on pain, fatigue, maximum oxygen uptake (VO_2_ max), body mass index (BMI), flexibility, and strength in patients with breast cancer.

Our first hypothesis is that the combined training reduces the pain in patients with breast cancer. The second one is that the combined training reduces the fatigue and BMI, besides increases the VO_2_ max, flexibility, and strength in patients with breast cancer. The last hypothesis is that the pain is related to the fatigue, BMI, VO_2_ max, flexibility, and strength.

## Methods

### Participants

Thirty-one female patients participated in this study; all were between the ages of 30 and 59, had not engaged in physical training over the previous 6 months, and were undergoing treatment (chemotherapy and radiation therapy) or being observed due to breast cancer at the Aldenora Bello Cancer Hospital (HCAB). This study excluded patients with mental or psychological disorders, those who were incapable of verbal communication or physical movement, and those who were pregnant or breastfeeding. Patients were excluded from the study if they missed three consecutive sessions, did not complete the evaluations, experienced psychological disturbances, became pregnant, quit, died, or were removed from the study by doctor’s orders.

Participants were informed about the objectives of the study and written informed consent was obtained. The study received approval from the Committee on Ethics in Research of the Federal University of Maranhão (UFMA), under protocol 20665713.2.0000.5087.

### Co-variables

Anthropometric measurements, such as weight (kg), height (cm) and age (years), were taken [11].

The marital status (single; married; widowed; divorced), educational level (high school; college), employment status (employed; unemployed), and family income (monthly income < twice the minimum wage; monthly income ≥ twice the minimum wage, where the minimum wage was $ 218.91) were ascertained by history-taking. Several hemodynamic variables were considered, such as the resting heart rate (HR), systolic blood pressure (SBP), and diastolic blood pressure (DBP) following resting in a comfortable position for 5 mins [11].

The type of tumor, pathologic stage of cancer (TNM classification = T: primary tumor; N: regional lymph nodes, M: distant metastasis) [25], the phase of treatment (chemotherapy, radiation therapy), and patient observation were evaluated through hospital records and patient histories.

The level of physical activity was assessed using the short version of the International Physical Activity Questionnaire (IPAQ). The participants were classified into the following: very active, active, occasionally active, and sedentary (those who did not engage in any physical activity for continuous 10 mins over the week) [34]. Each patient was also questioned for the duration she had stayed without participating in any physical training (3–12 months; more than a year; never participated).

### Primary outcome

The pain was assessed using the Brief Pain Inventory (Attachment A), validated for Brazilian Portuguese. This research tool evaluates not only the intensity and location of pain but also the degree to which pain interferes with a patient’s daily life and the effectiveness of pain management therapies; thus, the use of scales in hospital assessments can make it easier to identify and treat pain [10].

The questionnaire’s cut-off points are 4 for moderate pain and 8 for severe pain (1–4 = slight pain; 5–7 = moderate pain; and 8–10 = severe pain). Each score in the inventory varies from 0 (no interference or no pain) to 10 (the worst possible pain). The inventory is evaluated by looking at the numerical scores for each question; in other words, there is no general overall score for the questionnaire. In evaluating the dimensions, scores for each question in the inventory were averaged [10].

### Secondary outcomes

- Fatigue
  Fatigue was assessed using the Revised Piper Fatigue Scale (PFS-R), a validated instrument made up of 22 items distributed in three dimensions: behavioral, affective, and sensorial-psychological or sensorial-cognitive-emotional. Fatigue was then measured by averaging these three dimensions [26].
- Maximum oxygen uptake (VO_2_ max)
  The volume of VO_2_ max was determined using the American College of Sports Medicine (ACSM) submaximal cycle ergometer test [2]. The test based on a final force at a protocol of 15 W per minute, using the formula for women: *VO*_*2*_ *max (ml*^*− 1*^*Kg*^*− 1*^)* = 9.39 (measured in Watts) + 7.7 (body weight in kg) – 5.88 (age in years) + 136.7. * VO_*2*_ *max (ml*^*− 1*^*Kg*^*− 1*^ *min*^*− 1*^*) = Divided by Kg. Estimated standard error = 147 ml/min.*
- Body mass index (BMI)
  The BMI was obtained from the height and body mass using the formula BMI = mass (Kg) / height (m) ^2^ [2].
- Flexibility
  Flexibility was measured using the sit and reach test, which measures the flexibility of the hip joint as well as the lower back and hamstring muscles. The test used a Wells bench (Wells Portable Instant Pro Sanny) attached to a wall, where patients supported their feet, approximately at the width of their hips. With clasped hands, the patient stretches, reaching towards the bench as far as they can, without bending their knees or feeling pain. The furthest stretch out of three attempts was recorded as the measure of their flexibility [11].
- Strength
  Static strength was measured through the grip strength test, using a hand dynamometer (Jamar Sammons Preston), which has a scale from 0 to 100 k. Patients took the test by squeezing the dynamometer as tightly as they could without bending their elbow or altering their posture, in three repetitions, alternating their hands [11].

### Intervention

The combined training program consisted of a 12-week long course of aerobic, resistance, and flexibility exercises, with three sessions per week devoted to aerobic and resistance training in the same session (under the supervision of trainers specialized in physical exercise), and two sessions per week devoted to flexibility training (without supervision). The aerobic/resistance and stretching sessions took place on alternate days.

Each session of aerobic and resistance training lasted 60 mins, in the following order: 30 mins on the cycle ergometer (stationary exercise bicycle), hip flexion and extensions, shoulder exercises, squatting with a Swiss ball (stability ball), French presses (triceps extension exercises), and lifting exercises for the dorsal muscles. The flexibility training session lasted approximately 15 mins.

- Familiarization
  Participants were familiarized with the stretching exercises for 2 weeks, in three sessions per week, during this period each patient was coached on the correct performance of the stretching exercises. Familiarization with the aerobic and resistance training took place in three sessions over the course of a week, during which the cycle ergometer (stationary exercise bicycle) regimen was with 15 watts, and the resistance training regimen was with the patients’ body weight and a light Theraband, with 8–12 repetitions at one-minute intervals for each exercise.
- Aerobics
  Aerobic training was regulated using the target heart rate (THR) [18] from the following formula: *THR = x. (MHR – RHR) + RHR*, where x: % of the target effort, MHR: maximum heart rate, and RHR: resting heart rate. MHR was obtained using the cardiorespiratory test, and RHR was measured with the patient at rest. The THR was measured using a fitness monitor (Polar FT2).
  The cardiorespiratory fitness test was carried out using the ramp protocol [28] on a cycle ergometer (ERGO FIT brand, model ERGO 167-FITC CYCLE), starting with a five-minute warm-up at 15 watts, then raised by 15-watt increments at 60-s intervals. After reaching the maximum stage, there was a three-minute active recuperation period at the original 15 watts; the stages had between 70 and 90 rotations per minute (RPM). Every 15 s at the end of a stage, BP and HR measurements were obtained using a conventional sphygmomanometer (BD®) and Polar FT2, respectively. Perceived exertion was measured using the Borg Rating of Perceived Exertion scale (Infor Fisic). Before and after a session on the cycle ergometer, patients remained in a seated resting position while their BP and HR were measured, and their perceived exertion on the Borg scale was rated. The cardiorespiratory test was conducted 72 h after the familiarization.
  The rating of perceived exertion (RPE) was used to verify the intensity of individualized training (7–8 = Very easy; 9–10 = Easy; 11–12 = Somewhat easy; 13–14 = Slightly tiring; 15–16 = Tiring; 17–18 = Very tiring; and 19–20 = Exhausting), with patients verbally encouraged to reach their highest possible level of fatigue.
- Resistance
  The protocol for resistance training was three series of 12 repetitions for each exercise, with one-minute intervals between repetitions and series. Each movement was conducted at a speed of 3 s for the concentric phase and 3 s for the eccentric phase [5]. The exercises were alternated by segment, prioritizing the large muscle groups and employing ankle weights, weighted halters, elastic bands (Therabands), and the patient’s body weight.
  The training weight was determined by having the patient do 12 repetitions 72 h after familiarization [14]. Patients who were able to do more than 12 repetitions rested for 5 mins, and then repeated the 12 repetitions with additional weight.
- Flexibility
  The flexibility training involved active stretching (greater range of movement by a joint by contracting the agonist muscles and relaxing the antagonist muscles) without pain, where each exercise lasted for 20 s in three series [36]. The stretching exercises were: 1) Shoulder adduction with elbows extended, both sides; 2) Shoulder and elbow flexion with the palm on the back, both sides; 3) Fist flexion; 4) Fist extension; 5) Hip abduction with knees bent; 6) Hip flexion from a seated position with shoulder abduction and elbow flexion; 7) Touching the toes from a seated position with legs extended; 8) Touching the toes from a seated position with the legs extended and crossed, both sides; 9) Shoulder flexion and adduction with hands clasped together in front; and 10) Standing wall dorsiflexion.

- Weight progression
  Weight progressions were carried out every 4 weeks, respecting each patient’s individual biology in the cardiorespiratory fitness test and the maximum repetitions for predicting the initial weight [36]. Aerobic training began at 50–60% of the THR, ending at 80–90% of the THR. Resistance training began with the patient’s body weight or 1 kg in halters or ankle weights, and the moderate setting was used on the elastic band. In the fifth week, 1 kg was added to the weights, and the elastic band was increased to the high setting, where it was maintained until the twelfth week (Table 1).

**Table 1:** Weight progression in combined training of patients with breast cancer. São Luís, Maranhão, 2016

Weeks	Aerobic	Resistance
1 to 4	50–60% of THR	Body weight or 1 kg (halters and ankle weights), elastic band (Theraband) at medium setting
5 to 8	70–80% of THR	↑ 1 kg and elastic band (Theraband) at high setting
9 to 12	80–90% of THR	Maintain intensity from 5th to 8th week

*THR* Training Heart Rate

### Sample

The sample was for the convenience of breast cancer patients, and they were randomly assigned to intervention groups. Thirty-one patients participated in the research project, divided into a study group (SG) and a control group (CG).

### Calculation of the sample size

The sample of the pilot study was used for a sample inference of a controlled clinical trial in the Stata 10.0 program, using a test power of 80, 5% alpha, 1:1 division of groups in a matched pair test. The average pain of patients in the SG before the combined training (CT) was 4.79 ± 2.99; after the CT, it was 2.79 ± 2.22, yielding the results for a new study of 56 patients (SG = 28, CG = 28).

### Allocation

Patients were contacted and invited to participate in this study by telephone, through invitations issued at regularly-scheduled meetings with HCAB patients, and by referral from oncologists, mastologists, physiatrists, physical therapists, psychologists and pain management specialists. Patients who showed interest received a complete explanation of the study.

Groups were divided 1:1, with one additional patient in the CG. The groups were:

- SG, which underwent combined training (CT) for 12 weeks in addition to continuing their conventional hospital treatment (CHT) to breast cancer (chemotherapy, radiotherapy, and hormone therapy).
- CG, which underwent only CHT for 12 weeks and did not perform any physical training.

### Blind study

Assessments of both the SG and the CG were conducted at the study’s outset to establish a baseline, and at the end of 12 weeks, corresponding to the length of the combined training intervention. The team was trained in the application of each survey and test procedure, and the researchers were blinded with regards to the physical assessments, only being informed of the day and time of the assessments.

### Statistical methods

The Kolmogorov-Smirnov test was used to verify the normality of the variables. Variables were found to be normal for age, height, weight, HR, resting SBP and DBP, total points on the pain scale, the factor of pain intensity with regards to more, less, average and current pain, the factor of pain’s interference in the patient’s general activity, work, relationships, sleep, enjoyment of life, fatigue, VO_2_ max, BMI, flexibility, and static strength.

The matched pair student t-test was applied to the dependent and parametric variables, while the Wilcoxon matched pair test was applied to non-parametric and ordinal variables, and McNemar’s test was applied to paired and dichotomous variables. F test was used on independent parametric variables, and since all showed similar variations among the groups, the non-matched student t-test was applied. The Mann-Whitney test was used on independent non-parametric and ordinal variables, and the chi-squared test was applied to independent and dichotomous variables.

The secondary outcomes showed normal correlation with the pain intensity. The Pearson correlation test was used, with classifications being negligible (r = < 0.2), weak (*r* = 0.2–0.4), moderate (*r* = 0.4–0.6), strong (*r* = 0.6–0.8), and very strong (r= > 0.80) [12]. The statistical analyses were performed using the Stata 10.0 software, with α = 5%, meaning *p* < 0.05 considered as statistically significant.

## Results

**Fig. 1:**
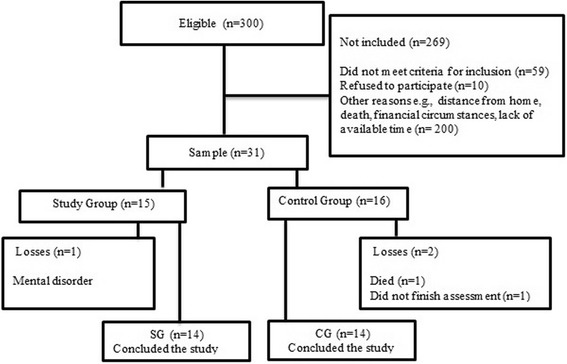
Sample flowchart. Study Group (SG); Control Group (CG)

Out of the 300 patients contacted, only 31 were willing to participate in the study. Of these, one patient had to be excluded from SG because of a diagnosis of mental disorder; in CG, one patient died, and another one failed to complete the final assessment. Overall, 28 patients completed the study (14 SG, 14 CG) (Fig. 1).

Compared with CG, patients in SG did not present significant differences in variables: anthropometric (age *p* = 0,5380, height *p* = 0,9026, weight *p* = 0,2028), marital status (*p* = 0,450), educational level (*p* = 1,000), employed (*p* = 0,139), household income (*p* = 0,686), hemodynamics (resting HR *p* = 0,3895, BPS at rest *p* = 0,6395, BPD at rest *p* = 0,1804), type of tumor (*p* = 0,0728), stage of illness (*p* = 0,9172), phase of treatment and observation (*p* = 0,3949), time since diagnosis (*p* = 0,2763), level of physical activity (p = 0,7291) and time since most recent physical training (*p* = 1,000). This shows homogeneity among the groups (Table 2).

**Table 2:** Anthropometric, social, and hemodynamic characteristics of patients with breast cancer (*n* = 28)

Variables	SG (*n* = 14)	CG (n = 14)	*p* value
Anthropometric^a^			
Age (years)	47,64 ± 7,60	45,79 ± 8,14	0,5380
Height (m)	154,77 ± 5,61	154,5 ± 6,01	0,9026
Weight (kg)	58,34 ± 9,29	64,57 ± 15,22	0,2028
Marital Status^b^			
Married	6(42,86%)	8(57,14%)	0,450
Single	8(57,14%)	6(42,86%)	
Educational level^b^			
High school	12(85,71%)	12(85,71%)	1000
College	2(14,29%)	2(14,29%)	
Employed^b^			
No	10(71,43%)	13(92,86%)	0,139
Yes	4(28,57%)	1(7,14%)	
Household income^b^			
< 2 Minimum wages	9(64,29%)	10(71,43%)	0,686
≥ 2 Minimum wages	5(35,71%)	4(28,57%)	
Hemodynamics^a^			
Resting HR (bpm)	81,86 ± 7	76,71 ± 12,10	0,3895
BPS at rest (mmHg)	114,57 ± 13,82	110,36 ± 11,57	0,6395
BPD at rest (mmHg)	73,21 ± 10,57	71,43 ± 9,33	0,1804
Type of Tumor^c^			
Ductal Carcinoma	14(100%)	11(78,57%)	0,0728
Fuso-cellular and Squamous cell	0	2(14,29%)	
Mixed tumor	0	1(7,14%)	
Stage of illness^c^			0,9172
0	1(7,14%)	0	
2	7(50%)	9(64,29%)	
3	5(35,71%)	4(28,57%)	
4	1(7,14%)	1(7,14%)	
Phase of Treatment and Observation^c^			
Observation	5(35,72%)	3(21,43%)	0,3949
Chemotherapy	6(42,86%)	8(57,14%)	
Radiation therapy	3(21,43%)	3(21,43%)	
Time since diagnosis^c^			0,2763
≤ 1 year	7(50.00%)	12(85.71%)	
1 the 5 years	5(35.71%)	1(7.14%)	
≥ 5 years	2(14.29%)	1(7.14%)	
Level of Physical Activity^c^			
Active	8(57,14%)	9(64,29%)	0,7291
Occasionally active	5(35,71%)	4(28,57%)	
Very active	1(7,14%)	1(7,14%)	
Time since most recent physical training^b^			
3 to 12 months	1(7,14%)	1(7,14%)	1000
> 1 year or never	13(92,86%)	13(92,86%)	

*HR* heart rate, *SBP* systolic blood pressure, *DBP* diastolic blood pressure; ^a^Non-paired Student T-Test; ^b^ Chi-squared test; ^c^ Mann-Whitney test; Expressed values: average ± standard deviation, absolute frequency (relative frequency)

Patients in SG displayed a significant reduction in total pain points (*p* = 0,0047), measurements of pain intensity (general intensity *p* = 0,0082, more *p* = 0,0284, less *p* = 0,0365 and average *p* = 0,0036) and the degree in which pain interfered in the patient’s life (general interfered *p* = 0,0201, mood *p* = 0,0252 and sleep *p* = 0,0499). Similar measurements in CG remained the same. There were no significant differences among the groups at the outset of the study or after 12 weeks (Table 3).

**Table 3:** Assessment of pain in patients with breast cancer (n = 28) who underwent physical training

Pain	SG (n = 14)			CG (n = 14)		
	Base	12 weeks	*p* value	Base	12 weeks	*p* value
Location of greatest pain^a^						
Head	0	1(7,14%)	0,8462	0	2(14,29%)	0,2995
Lower back	5(35,71%)	1(7,14%)		6(42,86%)	4(28,57%)	
Upper members	6(42,86%)	9(64,29%)		3(21,43%)	4(28,57%)	
Lower members	3(21,43%)	2(14,29%)		4(28,57%)	3(21,43%)	
None	0	1(7,14%)		1(7,14%)	1(7,14%)	
Total pain points^b^	4,29 ± 3,43	2,43 ± 3,76	0,0047*	3,43 ± 2,28	3,93 ± 4,41	0,6458
Treatment or medication^c^						
No	3(21,43%)	6(42,86%)	0,0833	6(42,86)	5(35,71%)	0,7055
Yes	11(78,57%)	8(57,14%)		8(57,14)	9(64,29%)	
Pain relief^a^	57,14% ± 41,96%	42,14% ± 49,33%	0,6319	63,57% ± 44,48%	43,57% ± 41,81%	0,4005
Intensity^b^	3,54 ± 2,49	2,46 ± 2,50	0,0082*	3.61 ± 3,03	3,82 ± 2,91	0,7175
More^b^	4,14 ± 2,88	2,86 ± 3,11	0,0284*	4,43 ± 4,11	4,57 ± 3,46	0,8469
Less^b^	2,79 ± 2,39	1,71 ± 1,86	0,0365*	2,07 ± 2,81	2,71 ± 2,40	0,3356
Average^b^	4,79 ± 2,99	2,79 ± 2,22	0,0036*	4,50 ± 3,67	4,57 ± 3,30	0,9449
Current^b^	2,43 ± 3,32	2,50 ± 3,30	0,5635	3,43 ± 3,76	3,43 ± 3,20	10,000
Interference^b^	3,54 ± 2,77	2,32 ± 3,05	0,0201*	4,12 ± 3,48	3,68 ± 3,82	0,6430
General activity^b^	3,93 ± 3,15	2,71 ± 3,20	0,0979	4,21 ± 4,23	4,14 ± 3,63	0,9420
Mood^a^	3,50 ± 2,77	1,50 ± 2,44	0,0252*	4,29 ± 4,45	3,14 ± 4,11	0,2365
Ability to walk^a^	3,71 ± 3,29	2,14 ± 3,48	0,0998	3,29 ± 3,65	3,57 ± 4,24	0,7467
Work^b^	3,79 ± 3,29	2,43 ± 3,27	0,0545	4,21 ± 4,35	3,57 ± 4,33	0,6290
Relationship^b^	3,07 ± 3,45	2,21 ± 3,45	0,1489	3,36 ± 3,82	3,50 ± 4,05	0,9087
Sleep^b^	3,79 ± 3,51	2,64 ± 3,03	0,0499*	5,71 ± 4,55	4,14 ± 4,15	0,2950
Enjoyment of life^b^	3,00 ± 3,44	2,57 ± 3,84	0,2256	3,79 ± 4,12	3,71 ± 3,93	0,9535

*SG* study group, *CG* control group, Expressed values: average ± standard deviation, absolute frequency (relative frequency); * = *p* < 0.05 (significant); ^a^ Wilcoxon Matched pair test and Mann-Whitney matched pair test; ^b^Matched pair and independent student t-test; ^c^McNemar and chi-squared tests

VO_2_ max (*p* = 0,0001), flexibility (*p* = 0,0001), and static strength (right *p* = 0,0001, left *p* = 0,0008) in both hands of participants in SG increased significantly, in contrast to those in CG. VO_2_ max also displayed a significant difference between the groups at the base (*p* = 0,0231). However, the difference was even greater after 12 weeks (*p* = 0,0001). However, fatigue (*p* = 0,0953) and BMI (*p* = 0,6088) were not significantly decreased in the SG (Table 4).

**Table 4:** Assessment of secondary outcomes in patients with breast cancer (n = 28) who underwent physical training

Variables^a^	SG (n = 14)			CG (n = 14)			SG X CG	
	Base	12 weeks	p value	Base	12 weeks	*p* value	Base	12 weeks
Fatigue	3,27 ± 3,03	2,27 ± 2,15	0,0953	3,85 ± 3,40	3,70 ± 3,07	0,7352	0,6371	0,1664
VO_2_ max (*ml*^*−1*^*Kg*^*−1*^ *min*^*− 1*^)	16,85 ± 1,94	20,68 ± 2,50	0,0001**	14,87 ± 2,39	14,80 ± 2,46	0,8359	0,0231*	0,0001**
BMI (Kg/m^2^)	24,30 ± 3,58	24,42 ± 2,86	0,6088	26,91 ± 5,46	26,89 ± 4,98	0,9581	0,1475	0,1197
Flexibility (cm)	18,86 ± 9,00	27,46 ± 7,25	0,0001**	25,07 ± 12,34	26,39 ± 12,47	0,2883	0,1399	0,7832
Static Strength (Kgf)								
Right	19,64 ± 7,08	24,79 ± 6,77	0,0001**	22,14 ± 7,42	21,71 ± 7,44	0,6566	0,3700	0,2636
Left	19,43 ± 6,58	22,71 ± 5,69	0,0008*	23,86 ± 6,54	23,29 ± 6,64	0,4533	0,0858	0,8087

*SG* study group, *CG* control group, *VO*_*2*_ *max* Volume of maximum oxygen uptake, *BMI* body mass index; Values expressed: average ± standard deviation; ^a^Matched pair and independent student t-tests *p < 0.05; **p < 0.01

Pain intensity showed a strong positive correlation with fatigue in SG at both the baseline measurement (*r* = 0,8571, *p* = 0,0001) and after 12 weeks (r = 0,6880, *p* = 0,0065), differently than the volume of maximum oxygen uptake, body mass index, flexibility, and static strength on the right and left sides for SG. Fatigue was also significantly correlated to pain intensity in CG (base r = 0,6511, *p* = 0,0117 and 12 weeks r = 0,7630, *p* = 0,0015) (Table 5).

**Table 5:** Correlation of pain intensity with secondary outcomes in patients with breast cancer (n = 28) who underwent physical training

Variables	SG (n = 14)				CG (n = 14)			
	Base		12 weeks		Base		12 weeks	
	r	*p* value	r	*p* value	r	*p* value	r	*p* value
Fatigue	0,8571	0,0001**	0,6880	0,0065*	0,6511	0,0117*	0,7630	0,0015**
VO_2_ max (*ml*^*−1*^*Kg*^*−1*^ *min*^*−1*^)	−0,1106	0,7066	−0,2123	0,4661	−0,4042	0,1518	−0,2182	0,4535
BMI (Kg/m^2^)	−0,3480	0,2227	−0,5858	0,0277*	−0,0920	0,7544	−0,1870	0,5221
Flexibility (cm)	−0,0217	0,9414	−0,0218	0,9409	− 03765	0,1846	−0,5575	0,0383*
Static Strength (Right) (Kgf)	−0,1358	0,6434	−0,2392	0,4102	−0,5810	0,0293*	−0,7252	0,0033**
Static Strength (Left) (Kgf)	−0,0339	0,9084	−0,3174	0,2688	−0,4673	0,0920	−0,5879	0,0270*

*SG* study group, *CG* control group, *VO*_*2*_ *max* Volume of maximum oxygen uptake, *BMI* body mass index, Values expressed: average ± standard deviation; *p < 0,05; **p < 0,01

## Discussion

There is no consensus in the literature regarding the ideal management of pain in patients with breast cancer; clinical studies that assess pain as an outcome in these patients are scarce [3, 6]. In this study, it was observed that patients with breast cancer who underwent 12 weeks of combined training, experienced a decrease in pain intensity, pain interference in their daily lives, and total pain points. These results corroborate with the observations in 25 patients undergoing treatment or clinical observation for breast cancer, who also experienced a decrease in pain. However, the combined training in that study was conducted in one aquatic session and six terrestrial sessions of self-massage, range of movement, strengthening, corrective measures, or as the patient preferred [23].

The combination of three types of different training in the present study may have contributed to the reduced pain in our group of patients with breast cancer. Aerobic exercises raise the peripheral levels of beta-endorphins, which reduces the sympathetic system activity, increases sleepiness, and produces psychological stability, in addition to improving the serotonergic system and the relation between nerve endings and the size of muscle fibers. Resistance exercises produce better synchronization of motor unit firings, more efficient motor unit recruitment, central nervous system activity, and motor-neuron excitability, in addition to depressing the inhibitory neural reflexes and inhibiting Golgi tendon organs [24, 35]. Finally, flexibility exercises produce better control over the articular structures and soft tissues [1].

In addition to reducing pain, the exercises in this study also increased VO_2_ max, flexibility, and strength. This demonstrates that physical training can be an effective non-pharmacological intervention during and after treatment for breast cancer.

One study showed an increase in cardiorespiratory fitness achieved through a 12-week aerobic training program [16]. This differed from a study that showed an increase in flexibility from an 8-week course of resistance and flexibility training [21]. Another showed an increase in static strength following 12 weeks of training in self-massage, a range of movement, corrective, and reinforcement exercises [23]. All of these studies were conducted with patients with breast cancer [16, 21, 23]. However, the latter two studies also showed a decrease in pain. This was in contrast with the first, in which pain was cited as a reason for giving up the treatment.

The increase in VO_2_ max with combined training may be due to the increase in cardiac output, and more significant interaction between alveolar ventilation and capillary blood flow, as well as skeletal muscles’ higher oxidative capacities, brought about by the exercises. The very low baseline readings for VO_2_ max among patients who did not participate in the combined exercise training group after 12 weeks is considered severe, since ventilation efficiency indices are adversely affected when VO_2_ max is less than 20 ml.kg^− 1^.min^− 1^, which is often the case for patients with severe cardiac insufficiency. Low VO_2_ max may be due to the toxicity caused by chemotherapy and radiation therapy, where hypoactivity in the parasympathetic system and hyperactivity in the sympathetic system activate the network of proinflammatory cytokines, which are present at high levels in patients with fatigue and cancer [9, 31].

The process of cachexia and sometimes weight gain may explain the inverse correlations between reduced flexibility and strength, and increased pain in patients who did not engage in physical training [29, 33].

No difference in BMI was observed among patients in this study, which corroborates the literature, such as a study that compared aerobic training with higher-intensity aerobic training and combined aerobic-and-resistance training [8], as well as another study that involved 10 weeks of walking activity. Both were successful at decreasing pain, but neither improved the BMI of patients with breast cancer [4].

Cancer patients can change their metabolism such as degradation of muscle mass, reduction in functional capacity, and loss of body fat. However, cancer treatment itself can cause the opposite, i.e., weight gain. Weight gain is also a function of aging; middle-aged women gain weight at the rate of 0.5 kg per year [29, 33]. Therefore, just the practice of exercising may not be sufficient to reduce BMI unless it is in combination with a healthy diet [15].

Although the proposed protocol did not reduce fatigue, this last one showed a significant positive correlation with pain intensity, both at the study’s outset and after 12 weeks for both groups. This symptom stems from some causes, such as psychological, social cognitive, behavioral and physical factors, as well side effects of radiation therapy, chemotherapy, and medications such as letrozole and exemestane used in hormone therapy [20, 30].

This study’s limitations include the indirect assessment of VO_2_ max and the small sample size. However, research projects focusing on patients during and after cancer treatment encounter difficulties with sample size because of adverse treatment effects and financial circumstances; this may contribute towards difficulty in patients getting to the locations where the physical training was offered [4]. The home-based training can be a modality that increases patients’ adhesion in future interventions because It allows the realization of the training in a variety of environments, which It is a possibility of inclusion for patients who live in distant places [22].

Nevertheless, with this small sample, we found that combined training was able to reduce pain and increase VO_2_ max, flexibility, and strength in patients with breast cancer. One of this study’s strong points was the use of combined training that—unlike other studies—included a 12-week course of aerobic, resistance, and flexibility exercises in five sessions per week; another strong point was the choice of pain management as a principal outcome, using a specific pain scale for cancer patients.

## Conclusion

This study demonstrated that combined training reduced total pain points, pain intensity, and interference of pain in patients’ daily lives, as well as increased maximum oxygen uptake, flexibility, and strength. However, no significant improvement was observed in fatigue or BMI for patients with breast cancers. Combined training that includes aerobic, resistance and flexibility exercises can be a useful aid to pain management for patients undergoing breast cancer treatment. Future research will be required to test the results observed here more efficiently.

## Abbreviations

ACSM: American College of Sports Medicine
BMI: Body Mass Index
BP: Bood Pressure
CG: Control Group
CHT: Conventional Hospital Treatment
CT: Combined Training
DBP: Diastolic Blood Pressure
HCAB: Hospital do Câncer Aldenora Bello
HR: Heart Rate
IPAQ: Physical Activity Questionnaire
Kg: Kilo
MHR: Maximum Heart Rate
PFS-R: Revised Piper Fatigue Scale
RHR: Resting Heart Rate
RPE: Rating of Perceived Exertion
RPM: Rotations Per Minute
SBP: Systolic Blood Pressure
SG: Study Group
THR: Target Heart Rate
TNM classification: T: primary tumor, N: regional lymph nodes, M: distant metastasis
UFMA: Federal University of Maranhão
VO2 max: Maximal Oxygen Uptake
